# Provisional Reduction Plating Versus External Fixation in the Staged Management of Gustilo–Anderson Type II–III Open Tibial Shaft Fractures

**DOI:** 10.3390/jcm14238421

**Published:** 2025-11-27

**Authors:** Yong-Cheol Yoon, Seok Hwan Yoon, Min Jun Kim, Hyoung-Keun Oh

**Affiliations:** 1 Orthopedic Trauma Division, Trauma Center, Gachon University College of Medicine, 21 Namdong-daero, 774 Beon-gil, Namdong-gu, Incheon 21565, Republic of Korea; dryoonyc@gmail.com (Y.-C.Y.); himman03@naver.com (M.J.K.); 2 Department of Orthopedic Surgery, Gachon University College of Medicine, 21 Namdong-daero, 774 Beon-gil, Namdong-gu, Incheon 21565, Republic of Korea; zxc2150@naver.com; 3 Department of Orthopedic Surgery, Inje University Ilsan Paik Hospital, 170 Juhwa-ro, Ilsanseo-gu, Gyeonggi-do, Goyang-si 10380, Republic of Korea

**Keywords:** open tibial fracture, intramedullary nailing, external fixation, reduction plating, infection, functional outcome

## Abstract

**Background/Objectives:** Open tibial shaft fractures are severe injuries associated with high risks of infection and malunion. Although external fixation is commonly used for provisional stabilization, it provides limited control over alignment and soft tissue handling. This study aimed to evaluate and compare provisional reduction plating with external fixation for the staged management of Gustilo–Anderson type II–III open tibial fractures. **Methods:** Fifty-nine patients (mean age, 38.5 years) treated with staged debridement and delayed intramedullary nailing (IMN) were retrospectively reviewed. Thirty-two patients underwent reduction plating with external fixation (group A), and 27 underwent external fixation alone (group B). Clinical, radiographic, and functional outcomes, including infection, union, malunion, operative time, and Lower Extremity Functional Scale (LEFS) scores, were analyzed. **Results:** Deep infection and nonunion rates were comparable between the groups. Group A had fewer malunions (3.1% vs. 18.5%), shorter operative times, and faster progression to union than group B. The LEFS scores were higher in group A, indicating better functional recovery. **Conclusions:** Provisional reduction plating before IMN appears to enhance alignment, preserve soft tissue integrity, and improve surgical efficiency without increasing the risk of infection or delaying union. These findings suggest that it may be a safe and effective adjunct to the staged treatment of complex Gustilo–Anderson type II–III open tibial fractures.

## 1. Introduction

Open tibial shaft fractures represent complex injuries that usually result from high-energy trauma and are frequently associated with severe soft tissue damage and comminution [1]. These injuries carry substantial risks of infection, malalignment, and nonunion [2]. Therefore, their management requires a staged, multidisciplinary approach that addresses both skeletal stability and soft tissue integrity.

The standard protocol typically involves urgent irrigation and debridement followed by provisional external fixation. Once the soft tissue envelope has improved, definitive fixation is most often achieved with intramedullary nailing (IMN) [3]. Although external fixation provides rapid stabilization, it is often insufficient for maintaining precise alignment in comminuted fractures. Furthermore, repeated intraoperative manipulations during IMN may prolong operative time and increase radiation exposure [4,5].

Adjunctive reduction plating was introduced to overcome these limitations [6]. This technique uses a small fragment plate, usually a 2.7 mm locking compression plate, applied through the traumatic wound after thorough debridement. The plate was intentionally left in place during subsequent IMN, and screw placement was carefully planned to avoid the medullary canal. This strategy maintains alignment during nailing, reduces the need for intraoperative manipulation, and may improve surgical efficiency [7,8].

However, some issues remain. The placement of additional internal hardware in open fractures has traditionally been viewed as problematic because of the risk of infection [9]. Implants in contaminated wounds may promote biofilm formation and compromise the periosteal blood supply, potentially increasing the risk of deep infection [10]. Furthermore, some authors have questioned whether combining a plate with an IMN, which is intended to provide relative stability, impairs bone healing, particularly in open fractures where soft tissue injury and periosteal disruption are already present [10]. Nevertheless, a recent report suggests that when meticulous debridement and surgical techniques are used, adjunctive reduction plating does not appear to increase infection rates and may enhance alignment control and promote union without additional morbidity [11].

Therefore, the present study aimed to compare the clinical and radiographic outcomes of Gustilo–Anderson types II–III open tibial shaft fractures treated with external fixation alone or with adjunctive reduction plating before definitive IMN. We hypothesized that reduction plating would improve the alignment and union without increasing the risk of infection.

## 2. Material and Methods

### 2.1. Study Design and Patients

This retrospective cohort study was conducted at two level I trauma centers. Medical records from January 2014 to December 2022 were reviewed to identify adult patients with open tibial shaft fractures who were treated using a staged surgical approach.

Patients were included if they were aged 18 years or older and had sustained a diaphyseal open tibial fracture classified as Gustilo–Anderson grade II or III [12]. All patients underwent staged surgical management, including initial external fixation followed by delayed definitive IMN. Patients with Gustilo–Anderson type I injuries, fractures treated with primary IMN, pathological fractures, periprosthetic fractures, Arbeitsgemeinschaft für Osteosynthesefragen/Orthopaedic Trauma Association (AO/OTA) system type 41 or 43 fractures, and those lost to follow-up before documented fracture union were excluded from the study.

A total of 59 patients met the inclusion criteria. The cohort included 41 men and 18 women (mean age, 38.5 years; range, 18–72 years; Table 1). The most common injury mechanisms were motor vehicle collisions (n = 25), motorcycle accidents (n = 14), falls from height (n = 11), and crush injuries (n = 9). The fractures were classified according to the AO/OTA system, with the majority being 42-A- and 42-B-type diaphyseal fractures [13]. The Gustilo–Anderson classification included 24 type II and 35 type III open fractures [12]. Polytrauma (Injury Severity Score ≥ 16) was present in 19 patients (32%).

All patients received initial management in the emergency department, including broad-spectrum intravenous antibiotics and splint immobilization. Surgical debridement and provisional fixation were performed within 24 h of injury in all cases.

### 2.2. Surgical Technique and Rehabilitation

#### 2.2.1. Group A: Provisional Miniplate and External Fixation (n = 32, Figure 1)

**Figure 1:**
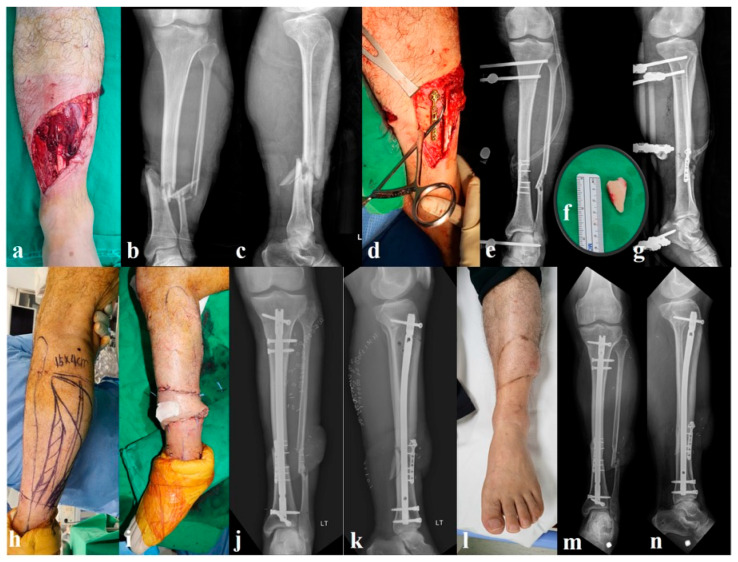
A 37-year-old man who sustained a Gustilo–Anderson type IIIB open tibial shaft fracture (Arbeitsgemeinschaft für Osteosynthesefragen/Orthopaedic Trauma Association 42-B) following a motorcycle accident (**a**–**c**). After irrigation and debridement, provisional reduction was achieved using a 2.7 mm miniplate (**d**) and stabilized with an external fixator (**e**). A free wedge fragment measuring 3 × 1.8 cm (**f**) was removed during debridement due to loss of viability. Definitive fixation with intramedullary nailing and rotational flap coverage was performed simultaneously (**h**–**k**). The wound healed uneventfully (**l**), and final follow-up radiographs showed complete bone union without additional bone grafting (**m**,**n**).

The initial surgery consisted of thorough irrigation and debridement of the open fracture wound, followed by the application of one or more miniplates to achieve provisional reduction of the tibia. The first was a 2.7 mm miniplate from the Compact Foot Set (DePuy Synthes, Oberdorf, Switzerland), and the second was a 2.8 mm miniplate for orthopedic trauma applications (Jeil Medical, Seoul, South Korea). These plates were contoured and applied to the exposed fracture surface [6]. Care was taken to minimize additional dissection, and plates were applied in areas already devoid of soft tissue (periosteum) due to the injury, avoiding further stripping. The plates were secured using bicortically placed screws whenever possible. Particular attention was paid to anticipate the path of the intramedullary nail, and all screws were intentionally directed to avoid this trajectory to prevent interference during definitive nailing. After plate fixation, a uniplanar external fixator was applied, spanning the tibia to provide additional stability. Wounds were irrigated again and primarily closed over drains when possible (in all type II and in most type IIIA cases) or covered with a vacuum-assisted closure dressing if there was a concern for soft tissue defects. A representative case is presented in Figure 1.

Definitive fixation was performed once soft tissue conditions were allowed (typically 1–2 weeks later). The external fixator was removed, and IMN of the tibia was performed in a standard manner. In cases of extensive soft tissue damage (e.g., Gustilo–Anderson type IIIB injuries), flap coverage was performed concurrently by the plastic surgery team at the time of definitive surgery. Notably, the provisional plates were not removed at the time of nailing; these were left in situ as a “permanent” reduction aid.

Postoperatively, patients in group A were typically encouraged to begin full weight-bearing as early as postoperative day 3. However, because these were open fractures frequently accompanied by soft-tissue swelling, wound care needs, and flap-related precautions, early mobilization was often not feasible. For this reason, the timing of weight-bearing was commonly adjusted in consultation with the plastic surgery team to accommodate soft-tissue recovery.

#### 2.2.2. Group B: External Fixation Alone (n = 27, Figure 2)

**Figure 2:**
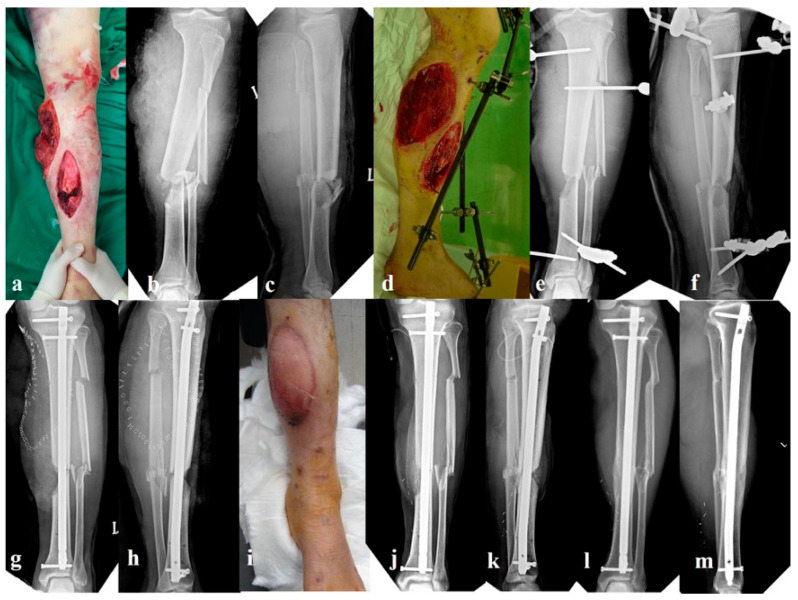
A 35-year-old man sustained a Gustilo–Anderson type IIIB open tibial shaft fracture (Arbeitsgemeinschaft für Osteosynthesefragen/Orthopaedic Trauma Association 42-B) following a traffic accident. Initial photographs and radiographs show a comminuted open fracture with extensive soft-tissue loss (**a**–**c**). After thorough irrigation and debridement, provisional stabilization was achieved using a uniplanar external fixator without application of a reduction miniplate (**d**–**f**). Definitive fixation with intramedullary nailing and rotational flap coverage was performed in the staged setting (**g**,**h**). After flap healing, autogenous bone grafting was performed to address the cortical defect (**i**–**k**). Final follow-up radiographs show solid bone union with satisfactory alignment and no signs of infection (**l**,**m**).

The initial management included thorough irrigation and debridement, followed by unilateral spanning external fixation without internal fixation. Primary wound closure was performed where feasible. Definitive IMN was performed after 1–3 weeks once the soft tissue conditions had sufficiently improved. At that time, the external fixator was removed, and fracture reduction was achieved manually before reamed nailing. Unlike group A, no provisional plates were used. Instead, reduction was achieved through traction and intraoperative manipulation, and in some cases, percutaneous reduction maneuvers using blocking pins were used to assist in maintaining proper alignment and rotational control during nail insertion. A representative case is presented in Figure 2.

Postoperative rehabilitation followed the same protocol as in group A. Although patients were encouraged to begin full weight-bearing from postoperative day 3, early mobilization was often difficult because of soft-tissue problems, so weight-bearing was delayed as needed.

### 2.3. Outcomes and Data Collection

We reviewed the patient charts, operative reports, and radiographs. The primary outcomes of interest were the following: infection rate, specifically deep infections (osteomyelitis or deep wound infections requiring surgical intervention); time to union, defined as the number of weeks from definitive surgery (nailing) to radiographic union; radiographic alignment and malunion rates. Radiographic union was determined by the presence of bridging calluses across at least three of the four cortices on biplanar radiographs along with the clinical absence of pain at the fracture site [14]. Nonunion was defined as failure to progress to union within 6 months or requiring additional intervention to achieve union [15]. Malunion was defined as an angulation > 5° in any plane or shortening > 1 cm at the union. Alignment was measured on postoperative and final follow-up radiographs; angular alignments in the coronal (varus-valgus) and sagittal (procumbent-recurvatum) planes were recorded [16].

The secondary outcomes included operative metrics (duration of the definitive nailing procedure) and functional recovery. The intraoperative fluoroscopy duration was also recorded as a part of the operative metrics. The C-arm screening time was measured in seconds from the first to the last image acquisition during definitive IMN and was retrieved from the device’s exposure log to ensure objective quantification. Functional outcomes were assessed using the Lower Extremity Functional Scale (LEFS) at the final follow-up (minimum, 12 months). The LEFS is a validated patient-reported outcome measure consisting of 20 items scored on a 0–4 scale (maximum score: 80), with higher scores indicating better lower limb function [17]. This was supplemented by the clinical assessment of joint range of motion, ambulatory ability, and return to work or activity. Return-to-work status was determined through outpatient follow-up interviews and a review of medical records. Patients were considered to have successfully returned to work or daily activities when they resumed their pre-injury occupational or functional levels without restriction for at least 4 consecutive weeks.

### 2.4. Statistical Analyses

Statistical analyses were performed to compare the clinical and radiographic outcomes between the two groups [18]. Continuous variables, such as time to union, operative time, and LEFS scores, were assessed for normality using the Shapiro–Wilk test. Variables with approximately normal distributions were compared using independent sample *t*-tests, and the results were presented as means with standard deviations. For variables that did not meet normality assumptions, non-parametric alternatives, such as the Mann–Whitney U test, were considered. Categorical variables, including infection rates, malunion rates, and return-to-work status, were analyzed using the chi-square test. When the expected cell count was less than 5, Fisher’s exact test was used to ensure statistical validity. All statistical tests were two-tailed, with the threshold for statistical significance set at *p* < 0.05. Statistical analyses were performed using SPSS version 26.0 (IBM Corp., Armonk, NY, USA).

## 3. Results

### 3.1. Demographics and Injury Characteristics

There were no statistically significant differences in the demographic or injury-related characteristics between the two groups (Table 1). Age and sex distributions were similar, and most injuries in both groups resulted from high-energy trauma. The severity of soft tissue injury, as classified by the Gustilo–Anderson system, was evenly distributed among the cohorts. Flap coverage was required in 12 cases in group A and in 10 cases in group B (rotational flap in 8 and 6 cases, respectively), with no significant intergroup difference (Table 2). Flap surgery was generally performed at the time of definitive fixation (“fix-and-flap”); when scheduling was not feasible, coverage was carried out as soon as possible thereafter. The mean interval from injury to definitive IMN was comparable between groups, averaging approximately 10 days.

### 3.2. Infection Outcomes

Six patients (18.8%) in group A and 4 patients (14.8%) in group B developed deep infections, consistent with the fracture-related infection (FRI) criteria (Table 3). Although most cases presented within the first 6 weeks post-injury and were initially managed with surgical debridement and antibiotics, 2 patients in group A and 1 patient in group B developed a late-onset infection (beyond 10 weeks post-injury). In accordance with international FRI treatment guidelines, these late cases required implant removal, extensive debridement, and refixation with new hardware to eradicate the infection and maintain stability. The remaining infections were managed using debridement, antibiotics, and implant retention, with good outcomes. All superficial infections resolved with oral antibiotics and none of the patients progressed to chronic osteomyelitis.

### 3.3. Fracture Union and Healing

Nonunion occurred in 6 patients (18.8%) in group A and in 5 patients (18.5%) in group B. All cases ultimately achieved bone healing after secondary procedures (Table 3). Two patients in each group were treated with exchange nailing, whereas the remaining patients required additional bone grafting. The mean time to union was 23.5 ± 4.8 weeks in group A and 26.7 ± 5.6 weeks in group B. Although this difference did not reach statistical significance (*p* = 0.08), there was a tendency toward faster healing in group A, which underwent provisional plating.

Radiographic analysis of the healing patterns revealed distinct differences between plated and non-plated cortices. The cortex opposite the plate generally showed callus formation, which is consistent with relative stability and indirect bone healing. In contrast, the plated cortex frequently demonstrated union without visible callus, reflecting absolute stability and direct bone healing. Thus, a mixed pattern of callus-mediated and direct cortical healing was observed in group A.

### 3.4. Radiographic Alignment and Malunion

Radiographic assessment revealed a more favorable alignment in group A. The mean coronal angulation was 1.8° ± 2.5° and the sagittal angulation was 2.1° ± 2.7° compared with 4.2° ± 3.5° and 4.8° ± 4.1° in group B, respectively. Although the differences in the alignment angles were not statistically significant, the lower values in group A suggested better anatomical restoration.

Malunion occurred in only 1 patient (3%) in group A, whereas 5 patients (18.5%) in group B experienced malunion (*p* = 0.08). Malunion occurred in 1 patient (3.1%) in group A and 5 patients (18.5%) in group B (*p* = 0.08). Although the difference in malunion rates was not statistically significant, the observed results suggest a potential clinical trend favoring group A. All malunion cases were managed conservatively and did not result in pain or functional deficits.

### 3.5. Operative Metrics

The mean operative time was significantly shorter in group A than in group B (71.2 ± 13.6 vs. 84.6 ± 19.8 min; *p* < 0.01). In group A, minor screw interference was encountered in two cases during nail insertion; these issues were resolved intraoperatively without altering the surgical plan.

In addition, fewer personnel are required in operating rooms. Traditionally, two assistants are required: one to control limb rotation and the other to assist the surgeon. With provisional plating in group A, rotational control was maintained, and a single assistant was sufficient. Furthermore, C-arm screening time was significantly reduced in group A compared with group B (95 ± 40 vs. 132 ± 55 s; *p* < 0.05), reflecting decreased need for intraoperative manipulation.

### 3.6. Functional Outcomes

At a minimum of 12 months’ follow-up, the LEFS score averaged 76.1 ± 6.7 in group A and 71.4 ± 5.2 in group B. Although the difference was not statistically significant (*p* = 0.09), it reflects a tendency toward improved functional outcomes in group A. Patients in group A resumed unrestricted weight-bearing earlier and demonstrated a higher rate of return to pre-injury activity levels (88% vs. 74%), but these differences did not reach statistical significance either. In addition to the LEFS, secondary outcomes included the clinical assessment of joint range of motion, gait status, and return-to-work rates, which showed no significant differences between the groups.

## 4. Discussion

This study evaluated whether provisional reduction plating before IMN offers clinical and radiographic advantages over temporary external fixation for the management of Gustilo–Anderson types II–III open tibial shaft fractures. Our findings demonstrate that provisional plating not only facilitates surgical efficiency, shorter operative times, and reduced fluoroscopy use but also appears to be associated with improved mechanical and functional outcomes [7]. Specifically, patients in the plating cohort experienced fewer malunions, slightly faster progression to union, and better patient-reported function, as reflected in the higher LEFS scores. Although these differences did not reach statistical significance, the observed trends suggest potential clinical benefits of provisional plating. Importantly, these benefits were achieved without a significant increase in infections or other complications, thus addressing one of the primary concerns traditionally associated with internal fixation in open fractures [19]. Taken together, these results suggest that provisional plating can fulfill the dual aim of improving alignment and healing while maintaining a safety profile comparable to that of external fixation.

In our cohort, deep infection developed in 18.8% of patients treated with provisional plating and 14.8% of those managed with external fixation alone, with no statistically significant difference (*p* = 0.74). Although the plating group demonstrated a slightly higher rate, the absolute difference was small and did not suggest a clinically significant disadvantage. These results are in line with contemporary studies indicating that infection risk is driven more by the severity of open injury and quality of debridement than by the provisional fixation method [9,20]. Historical reports, such as the classic trial by Bach and Hansen, cautioned against internal fixation in open tibial fractures because of higher osteomyelitis rates [21]. However, more recent evidence, including studies by Ludwig et al. [7] and Revak et al. [11], supports that temporary or even permanent plates, when used within a staged protocol, do not significantly increase infection risk. Our findings suggest that provisional plating can be safely incorporated into staged management strategies without increasing the risk of deep infections.

Provisional plating in our series was associated with a low rate of malunion, with only 1 case (3.1%) compared to 5 cases (18.5%) in the external fixation group (*p* = 0.08). This finding highlights the advantage of using a plate to achieve anatomical reduction and maintain alignment during the interim [8]. Even with a small fragment plate, provisional fixation helps restore length, rotation, and angulation, thereby reducing the need for extensive manipulation during definitive IMN [22]. Importantly, plating facilitates soft tissue management. Stable reduction and temporary fixation of fragments allow for easier primary repair of the open wound and minimizes tension across the soft-tissue envelope [23]. In contrast, external fixation alone often struggles to control fracture alignment with the same precision, which can lead not only to subtle deformities but also to increased wound tension and a higher risk of necrosis. These combined mechanical and biological benefits likely contributed to the lower malunion rate and more favorable wound conditions observed in the plating cohort.

Importantly, the use of a provisional plate did not delay the fracture healing. The average time to union was slightly shorter in the plating group (23.5 weeks) than in the external fixation group (26.7 weeks), but this difference was not statistically significant (*p* = 0.08). This suggests that the additional dissection required for plate application did not compromise the biology or slow down the healing process. Our observation is in line with a report by Revak et al., who also found no meaningful difference in union times when a reduction plate was used as part of the staged management for open tibial fractures [11]. Nonunion occurred at comparable rates in both groups (18.8% with plating vs. 18.5% with external fixation, *p* = 1.00). Although secondary procedures such as exchange nailing or bone grafting are sometimes required, all patients ultimately achieve union. Taken together, these results indicate that provisional plating offers the benefit of improved fracture alignment without the risk of delayed union or nonunion, reinforcing the technique as a biologically safe option within a staged treatment strategy [6].

Another important strength of this study is its design. Unlike many previous reports that combined closed and open fractures or included heterogeneous injury types, our investigation focused exclusively on Gustilo–Anderson types II–III open tibial shaft fractures [2,12,20]. Furthermore, all patients were treated with a consistent staged protocol: initial debridement and provisional fixation, followed by delayed IMN, which reduced heterogeneity and enhanced the reliability of the comparisons. A unique feature of our protocol is that provisional miniplates are deliberately left in place during definitive nailing. This decision was based on biological and practical considerations. Reamed debris provides an autologous bone graft effect that is especially valuable in open fractures, and reopening the wound for plate removal would risk losing this benefit while causing additional soft tissue injury. Importantly, the retained plates did not interfere with nail placement or compromise bone healing; instead, they contributed to stable alignment without adverse effects.

In our cohort, the plating group did not differ significantly from the external fixation group in terms of flap requirements or the overall complexity of soft tissue management. The use of a provisional miniplate did not increase the need for flap coverage or complicated wound care. However, an open wound with exposed bone fragments and a plate in place is not ideal. To minimize contamination risk, irrigation and debridement were performed every 48 h until coverage was achieved [4]. When flap surgery was required, we coordinated closely with the plastic surgery team so that definitive fixation and soft tissue reconstruction could be performed at the earliest opportunity (Figure 1). This approach reflects recommendations from previous studies that emphasize that provisional plating should never delay timely coverage and must be coupled with meticulous wound care [3,9,10]. Our experience supports this view, underscoring that the success of provisional plating relies on rigorous debridement protocols and close collaboration between reconstructive surgeons.

However, provisional plating is not universally applicable. In cases of extensive soft tissue loss, severe contamination, or a high risk of infection, the addition of internal hardware may be impractical or even hazardous. Moreover, this technique requires additional surgical time, specialized implants, and careful coordination with the reconstruction teams, which may not always be feasible in resource-limited settings. These practical considerations suggest that provisional plating should be viewed as a selective adjunct, rather than a universal solution, for all open tibial fractures.

This study has several limitations. The retrospective design and modest sample size reduce the statistical power of our analyses. Given the limited sample size and retrospective design, the study may have been underpowered to detect certain differences between groups, particularly those with marginal *p*-values, and some non-significant findings should therefore be interpreted with caution. For example, based on our post hoc estimation, detecting the observed difference in malunion rates (3.1% vs. 18.5%) with 80% power at α = 0.05 would require a considerably larger cohort, likely involving several dozen additional patients per group.

There is also a potential selection bias; surgeons may have opted for external fixators in the most severe injuries or when concern for infection was highest, which could inflate the complication rates in that cohort. We attempted to control for injury severity, and the baseline fracture characteristics were similar between the groups; however, unmeasured confounders could still have influenced the results. Because this study was conducted across two institutions, the possibility of intra-site correlation could not be completely ruled out. Although we explored including site as a covariate, the limited number of clusters precluded more robust mixed-effects modeling. Future multi-center studies with larger sample sizes are needed to validate these findings.

The follow-up duration was variable, and some patients were lost after healing, potentially affecting the assessment of long-term outcomes like function. Although the LEFS is a bounded scale (0–80), it was treated here as a continuous variable based on its approximate normal distribution in our sample. Future studies may consider more complex statistical models (e.g., logistic or beta regression) to better account for the bounded nature of such outcome measures. Another consideration is that the definition of malunion can vary. We defined malunion as any angulation > 5° or rotation > 10° at the union, which may not capture subtle functional malalignments. Nonetheless, none of the patients required revision for malalignment, suggesting that the residual deformities were minor.

## 5. Conclusions

Provisional reduction plating prior to IMN in types II–III open tibial shaft fractures was associated with a tendency toward improved alignment, fewer malunions, and a trend toward improved functional recovery compared with external fixation alone, without increasing infection or other complications. These findings support provisional plating as a safe and effective adjunct in the staged treatment of complex open tibial fractures and suggest that these observed trends may be clinically meaningful. Future studies with larger samples are needed to confirm these results and determine their generalizability.

## Figures and Tables

**Table 1:** Patient demographics and injury characteristics.

Variable		Group A (n = 32)	Group B (n = 27)	*p*-Value
Age (years)		37.5 ± 12.4	39.1 ± 11.7	0.61
Male		22 (68.8)	19 (70.4)	0.89
Previous medical history	HTN	7	6	0.64
	DM	4	5	
	Others	6	3	
Smoking history		13	11	1.00
Injury mechanism	MVC	14	11	0.88
	Motorcycle	8	6	
	Fall	6	5	
	Crush	3	4	
	Others	1	1	
Gustilo–Anderson classification	II	12	12	0.96
	IIIA	10	8	
	IIIB	7	5	
	IIIC	2	2	
AO/OTA classification	42-A	18 (56.3)	16 (59.3)	0.89
	42-B	10 (31.3)	7 (25.9)	
	42-C	4 (12.5)	4 (14.8)	
Polytrauma (ISS ≥ 16)		9 (28)	10 (37)	0.58
ASA score	I	5	4	0.92
	II	15	14	
	III	10	8	
	IV	2	1	

Values are presented as mean ± standard deviation, number (percentage), or number. Group A, provisional plating and external fixation; group B, external fixation only; DM, diabetes mellitus; HTN, hypertension; MVC, motor vehicle collision; AO/OTA, Arbeitsgemeinschaft für Osteosynthesefragen/Orthopaedic Trauma Association; ISS, Injury Severity Score; ASA, American Society of Anesthesiologists.

**Table 2:** Perioperative course and surgical efficiency.

Variable		Group A (n = 32)	Group B (n = 27)	*p*-Value
Time from injury to first I&D (hours)		10 [2–21]	11 [2–18]	0.48
External fixator duration before IMN (days) ^1^		10 ± 5	12 ± 7	0.22
Operative time at IMN (minutes)		71.2 ± 13.6	84.6 ± 19.8	<0.05
C-arm screening time (seconds)		95 ± 40	132 ± 55	<0.05
Soft-tissue coverage within 7 days ^1^		25 (78.1)	20 (74.1)	0.77
Coverage method ^1^	Local/rotational	8	6	—
	Free flap	4	4	
IV antibiotic duration (days) ^1^		11 ± 4	10 ± 4	0.34

Values are presented as median [interquartile range], mean ± standard deviation, or number (percentage), or number. ^1^ These variables were added based on literature evidence or institutional practice. Group A, provisional plating and external fixation; group B, external fixation only; I&D, irrigation and debridement; IMN, intramedullary nailing; IV, intravenous.

**Table 3:** Radiographic and functional outcomes and complications.

Variable	Group A (n = 32)	Group B (n = 27)	*p*-Value
Time to union (weeks)	23.5 ± 4.8	26.7 ± 5.6	0.08
Coronal angulation at union (°)	1.8 ± 2.5	4.2 ± 3.5	0.07
Sagittal angulation at union (°)	2.1 ± 2.7	4.8 ± 4.1	0.06
LEFS at final follow-up	76.1 ± 6.7	71.4 ± 5.2	0.09
Time to protected WB (weeks) ^1^	4.0 ± 2.5	4.8 ± 1.8	0.11
Deep infection (FRI 2018 definition) ^1^	6 (18.8)	4 (14.8)	0.74
Superficial infection	1 (3.1)	2 (7.4)	0.59
Nonunion	6 (18.8)	5 (18.5)	1.00
Malunion (>5° or >2 cm)	1 (3.1)	5 (18.5)	0.08
Pin-site infection while in external fixation ^1^	3 (9.4)	2 (7.4)	1.00
Reoperation (any cause) ^1^	12 (37.5)	9 (33.3)	0.79
Exchange nailing for nonunion	2	2	—
Bone graft for nonunion	4	3	
DAIR (for FRI) ^1^	4	3	—

Values are presented as mean ± standard deviation, number (percentage), or number. ^1^ These variables were added based on literature evidence. Group A, provisional plating and external fixation; group B, external fixation only; LEFS, Lower Extremity Functional Scale; WB, weight-bearing; FRI, fracture-related infection (2018 definition); DAIR, debridement, antibiotics, and implant retention.

## Data Availability

The datasets generated and/or analyzed during the current study are not publicly available because of restricted access to our hospital database but are available from the corresponding author upon reasonable request.

## Abbreviations

The following abbreviations are used in this manuscript:

IMN	Intramedullary nailing
LEFS	Lower Extremity Functional Scale
AO/OTA	Arbeitsgemeinschaft für Osteosynthesefragen/Orthopaedic Trauma Association
FRI	Fracture-related infection
